# FlexLMM: a Nextflow linear mixed model framework for GWAS

**DOI:** 10.1093/bioinformatics/btaf021

**Published:** 2025-01-15

**Authors:** Saul Pierotti, Tomas Fitzgerald, Ewan Birney

**Affiliations:** European Molecular Biology Laboratory, European Bioinformatics Institute (EMBL-EBI), Cambridge CB10 1SD, United Kingdom; European Molecular Biology Laboratory, European Bioinformatics Institute (EMBL-EBI), Cambridge CB10 1SD, United Kingdom; European Molecular Biology Laboratory, European Bioinformatics Institute (EMBL-EBI), Cambridge CB10 1SD, United Kingdom

## Abstract

**Summary:**

Linear mixed models (LMMs) are a commonly used statistical approach in genome-wide association studies when population structure is present. However, naive permutations of the phenotype to empirically estimate the null distribution of a statistic of interest are not appropriate in the presence of population structure or covariates. This is because the samples are not exchangeable with each other under the null hypothesis, and because permuting the phenotypes breaks the relationship among those and eventual covariates. For this reason, we developed FlexLMM, a Nextflow pipeline that can perform appropriate permutations in LMMs while allowing for flexibility in the definition of the exact statistical model to be used. FlexLMM can set a significance threshold via permutations, thanks to a two-step process where the population structure is first regressed out, and only then are the permutations performed on the uncorrelated residuals. We envision this pipeline will be particularly useful for researchers working on multi-parental crosses among inbred lines of model organisms or farm animals and plants.

**Availability and implementation:**

The source code and documentation for the FlexLMM is available at https://github.com/birneylab/flexlmm.

## 1 Introduction

Linear mixed models (LMMs) are a well-established approach for Genome-Wide Association Studies (GWAS) (Alamin *et al.* 2022). They can effectively correct for population structure by modelling the covariance of the phenotypes as a function of a genetic relatedness matrix. They can also improve power by implicitly conditioning on loci other than the one being tested, and can be used for estimating the heritability of a phenotype (Yang *et al.* 2014). Several efficient implementations of LMMs specific for GWAS have been developed. Most notably BOLT-LMM (Loh *et al.* 2015), fastGWA (Jiang *et al.* 2019), SAIGE (Zhou *et al.* 2018), and GCTA (Yang *et al.* 2011). A different approach but with similar goals is the whole-genome regression implemented in REGENIE (Mbatchou *et al.* 2021). These software are highly optimized for handling large human datasets such as the UK Biobank (Sudlow *et al.* 2015). However, this optimization comes at the price of reduced flexibility in the specification of the statistical models to be compared for evaluating significance. Moreover, in organisms where a community-accepted genome-wide significance threshold is not available, sample permutations are a “gold standard” method for avoiding false positives, but this approach is not valid for LMM if naively implemented (Joo *et al.* 2016).

**Figure 1.**
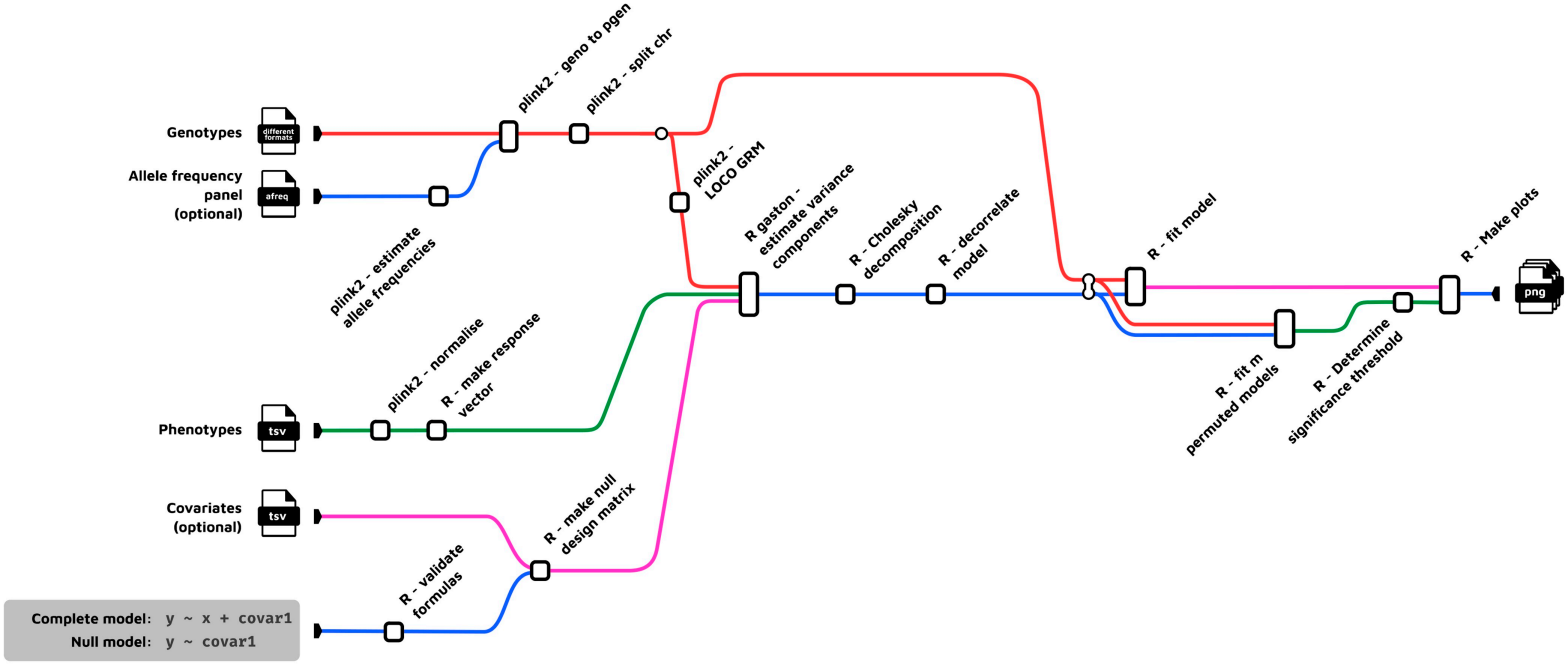
Simplified overview of the FlexLMM pipeline. The flow of information is from left to right. Items on the left represent input files or values. Items on the right represent output files. Not all the output and input options and formats are shown.

We developed FlexLMM as a solution for these two issues. FlexLMM can take in input an arbitrary statistical model for the fixed terms (e.g. it is possible to modify the genotype encoding to account for dominance, or to add a gene-by-environment interaction term), and compares it to an arbitrary null model via a likelihood ratio test. In addition, FlexLMM can natively run permutations to define a genome-wide significance threshold. The main issue with permutations in LMMs is the fact that the samples are not exchangeable under the null hypothesis (e.g. the samples are not all equally related to each other). To address this issue, FlexLMM first estimates the variance–covariance structure from the original datasets, and then regresses it out from the phenotype and design matrix. The residuals are evaluated under the null model in this uncorrelated space and permuted by taking their interdependence into account, defining a new shuffled phenotype vector. After multiple permutations, this process empirically defines a null *P*-value distribution that allows for the selection of an appropriate genome-wide significance threshold. Our permutation approach is almost equivalent to the one used in MVNpermute (Abney 2015), with the difference that we do not perform a back-projection of the residuals to the original space. Instead, we remain in the uncorrelated space where we can immediately define the null *P*-value distribution using Ordinary Least Squares (OLS). Permutation approaches comparable to the one we adopted were also followed in recent work (Scott *et al.* 2021, John *et al.* 2022, 2024) (Fig. 1).

## 2 Implementation

The FlexLMM logic is coded in the Nextflow Domain Specific Language version 2 (DSL2) (Di Tommaso *et al.* 2017), while individual steps are mainly taking advantage of the excellent plink2 suite of tools (Chang *et al.* 2015) and of the R programming language (R Core Team 2023) (https://www.r-project.org/). Other packages that are used in the pipeline are gaston (Claire and Hervé 2018), ggplot2 (Wickham 2016) (https://ggplot2.tidyverse.org/), cowplot (Wilke 2020) (https://wilkelab.org/cowplot/), and ComplexHeatmap (Gu *et al.* 2016). Moreover, we use containerization solutions in all the pipeline steps, allowing for complete reproducibility of computational workflows. For routine genetic data handling (construction of genetic relatedness matrices, format conversions, slicing and parsing genotype matrices) we use the plink2 suite because of its highly optimized code and file formats. For more custom operations, such as the definition of design matrices and contrasts, and model fitting and permutation, we use our own R code. The use of the Nextflow language in FlexLMM allows for a high degree of task parallelization and seamless portability to a wide range of computational platforms with minimal effort on the part of the user. The pipeline is designed to run different chromosomes, phenotypes, and permutations in parallel as separate Nextflow processes.

The user is required to provide a file containing the genotypes of interest (in VFC, BCF, plink, or bgen format), and tab-separated files containing sample-specific values for phenotype (optionally also multiple phenotypes to be run in parallel) and covariates. For the moment FlexLMM supports only quantitative phenotypes. The user needs also to provide the statistical model to be used for association testing in the form of an R formula, and a null model (also as an R formula) to be used for estimating heritability and for testing significance via a likelihood ratio test.

As a design choice, no imputation of missing values is performed in FlexLMM. Any missing values that are present in the data supplied to FlexLMM will cause the affected samples to be excluded from the analysis. If a different treatment of missing values is desired, the user should perform any imputation process upstream of running FlexLMM.

The pipeline implements an LMM approach to Genome-Wide Association Studies (GWAS), which takes the form:


y=Xb + Zu + ε,

where the *n × 1* vector of phenotypes *y* is modelled as a function of a design matrix *X* of dimensions *n × q* of variables treated as fixed effects, that multiplies a *q × 1* vector *b* of fixed effect coefficients. The random effect terms *Zu* are similarly composed of a *n × p* design matrix *Z* and a random effects vector *u* of dimensions *p × 1*. The residual vector *ε* is of dimensions *n × 1* and follows a multivariate normal distribution $N(0,\sigma_{\varepsilon }^{2}\;I)$ with spherical covariance. The random effects *Zu* are also modelled with a multivariate normal distribution $N(0,\sigma_{g}^{2}\;K)$, where $K=ZZ^{T}$ is the Genetic Relatedness Matrix (GRM).

In FlexLMM, the GRM is computed in a Leave-One-Chromosome-Out (LOCO) fashion, so as to avoid double-fitting the same variants as both fixed and random effects. The variance components ${\sigma_{\varepsilon }}^{2}$ and $\sigma_{g}^{2}$ are estimated using the lmm.aireml function of the gaston package, using only nongenetic fixed effects (more specifically, the design matrix corresponding to the user-defined null model is used). The residual variance–covariance matrix is then evaluated as $\Sigma =\sigma_{g}^{2}\;K+\sigma_{\varepsilon }^{2}\;I$, and regressed out from the phenotype vector and the design matrix of fixed effects using a Cholesky decomposition $\Sigma =LL^{T}$. The transformed design matrix $X_{mm}=L^{-1}\;X$ and response vector $y_{mm}=L^{-1}\;y$ can then be used in a simple linear regression to estimate fixed effects. Fixed effects are estimated separately for the null model design matrix that does not include any genetic variable, and then for the model including the genetic variant of interest (or a function of it). The model including the genetic variant of interest is fit as many times as there are genetic variants to be tested, using always the same variance components for the decorrelation step. This has the advantage of requiring only one fit for the variance components per chromosome.

The use of user-defined models, that can be specified as pipeline parameters using the R formula interface, allows for maximum flexibility. The formulas can use a special term “x,” which is understood by the pipeline to refer to the current genetic variant of interest. This allows for fitting more complex models that can include nonlinearities, such as a dominance term or gene-by-environment interactions. Statistical significance is determined by performing a likelihood ratio test between the null model and the complete model defined by the user.

To account for multiple testing, the pipeline can perform permutations of the residuals after the decorrelation step. This makes the errors from the model uncorrelated, and so exchangeable under the null hypothesis of no effect due to genetic variants. However, despite the true errors ϵ being independent the realized residuals $e_{mm\;}=y_{mm}-X_{mm}\;\hat{b}$ are not yet exchangeable due to the loss of degrees of freedom consequent to the fixed-effect estimation. This interdependence can be removed by projecting the residuals to a lower-dimensional space before performing the permutations. The realized residuals $e_{mm}\;$have covariance structure $V=I-X_{mm}\;{\left(\;X_{mm}^{T}\;X_{mm}\;\right)}^{-1}\;X_{mm}^{T}$. The spectral decomposition $V=U\Lambda U^{T}$ has the first *n–q* eigenvalues equal to 1 and the remaining *q* eigenvalues equal to 0, with *n* being the number of samples and *q* the number of fixed effect columns. By forming a matrix $U_{1}$ of eigenvectors corresponding to the nonzero eigenvalues, it is possible to project the residuals $e_{mm}$ to a *n–q* dimensional space where they are exchangeable under the null hypothesis. The new uncorrelated residuals $\zeta_{mm}=U_{1}^{T}\;e_{mm}$ are subsequently permuted and the permuted version $\zeta_{mm}^{\pi }$ is projected back to the sample space as $e_{mm}^{\pi }=U_{1}\;\zeta_{mm}^{\pi }$. A new shuffled phenotype is defined as $y_{mm}^{\pi }=X_{mm}\;\hat{b}+e_{mm}^{\pi }$, and can be used in a regular linear regression model $y_{mm}^{\pi }=X_{mm}\;b+\epsilon$ to define a null *P*-value distribution.

Compared to a permutation of the genotypes or phenotypes, this approach has the advantage of maintaining the 3-way relationship between phenotypes, genotypes, and covariates that is present under the null hypothesis. For a more detailed description of this approach, we refer the reader to (Abney 2015). The permutations are repeated multiple times (defined by the user), and the minimum *P*-value obtained in each permutation across the genome is stored. The set of minimum *P*-values is used to define the null distribution for genome-wide significance.

## 3 Conclusion

We have developed FlexLMM, a Nextflow pipeline that can run LMMs for genome-wide association studies. The key advantage of our implementation is the flexibility in the definition of the exact statistical model to be used, and the native implementation of permutations for the definition of an empirical significance threshold. These features will make the pipeline particularly useful for scientists working with structured nonhuman populations, such as multi-parental F2 crosses among inbred lines of model organisms or farm animals and plants. The implementation using the Nextflow language allows for extreme portability to a variety of computational infrastructures and automatic parallelization of tasks, with minimal installation and tuning effort on part of the user. The pipeline can natively test for Gene by Environment interactions (GxE), and epistatic interactions (GxG) against predetermined genetic variants by simply specifying the appropriate model and null model. We also plan to add support for binary phenotypes and expression Quantitative Trait Loci (eQTLs) in a future version of the pipeline.

Conflict of interest: None declared.

## Data Availability

No new data was used.
